# Maintaining social care provision in the context of financial austerity

**DOI:** 10.23889/ijpds.v3i1.585

**Published:** 2018-11-12

**Authors:** S Chotvijit, M Thiarai, S Jarvis

**Affiliations:** 1 Warwick Institute for the Science of Cities, University of Warwick, Coventry, United Kingdom; 2 Birmingham City Council Birmingham, United Kingdom

**Keywords:** Birmingham, Authority, Services, Safeguarding, Analytics, Data

## Abstract

There is significant national interest in tackling issues surrounding the needs of vulnerable children and adults. At the same time, UK cities are under significant financial strain, as local government financial settlements (the distribution of central government resources) decrease in real terms and yet urban populations, which draw on local government services, continue to grow. This study focusses on the city of Birmingham, the UK’s largest and most populous city outside of London. In a data-led study, using data derived from personal social care records, we analyse the management and delivery of social care services by Birmingham City Council, which itself is the largest local authority in Europe. This research employs state-of-the-art data analytic techniques to analyse six years of Birmingham City Council social care data, to identify: (i) Service cost profiles over time; (ii) Geographic dimensions to service demand and delivery; (iii) Patterns in the provision of services, which may assist with future service planning and provision and (iv) The extent to which data value and data protection interact. In response to recent fiscal challenges, Birmingham City Council is expected to make savings of £815 million over the 9-year period 2011/12 to 2019/20. Delivering savings of this scale, whilst protecting and safeguarding the most vulnerable citizens within a growing urban population, is one of the biggest challenges facing the UK’s second largest city.

## Introduction

### Birmingham and its City Council

The city of Birmingham is the UK’s largest and most populous city outside of London. Birmingham has a population of over 1.1 million people, and the population is growing faster than the UK average [1]. Birmingham is a young and diverse city; half of the population are aged 30 or under, and the city benefits from many different nationalities, faiths, languages, ethnicities and cultures.

Birmingham faces many challenges. Birmingham is the sixth most deprived local authority in the UK; 40% of the city is ranked in the most deprived 10% of areas in England. There are significant levels of child poverty; 30% of the city’s children live in a deprived household [2]. Life expectancy is worse in Birmingham than the average found across the remainder of England. Life expectancy also varies significantly between the most and least deprived areas (7.6 years lower for men and 6.2 years lower for women).

**Figure 1: Birmingham and its 10 council constituencies and 40 electoral wards:**
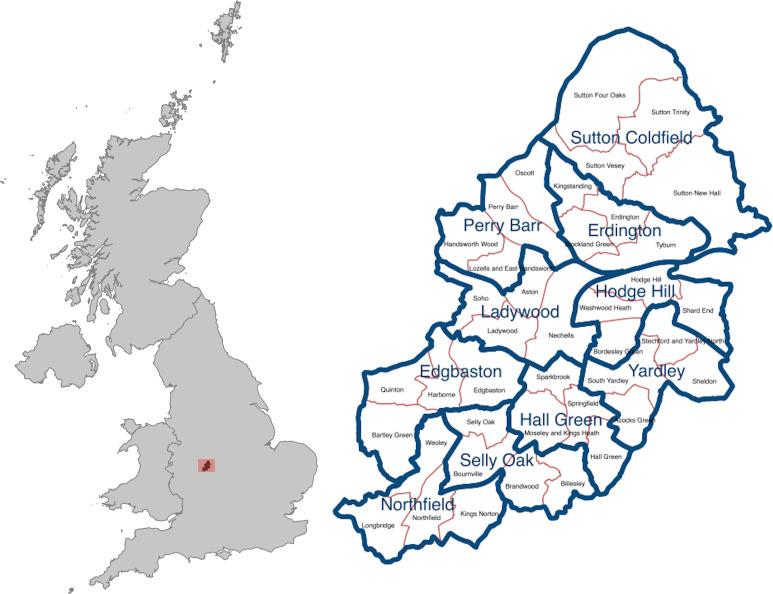


Birmingham City Council (BCC) is the local government body responsible for the governance of the city, which is managed through the division of the city into 10 council constituencies and 40 electoral wards, see Figure 1. BCC is the largest local authority in Europe. Income and expenditure in 2016/17 was £3.094 billion, of which £782 million was spent on schools, £550 million spent on benefits, £805 million spent on services for people and £287 million spent on housing [3]. Managing BCC’s priorities - including maximizing the independence of adults, sustaining neighbourhoods, and growing the economy and jobs - has been challenging in the context of recent fiscal challenges. Birmingham City Council is expected to make total savings of £815 million over the 9-year period 2011/12 to 2019/20 and, as a result of this, BCC is expected to reduce staff from 20,000 in 2010 to around 7,000 by 2018 [2].

### City Analytics

This research was established in the context of a reduction in workforce and proposed further cost savings in social care in Birmingham. In addition, in 2010, 2012 and 2014, the provision of social services to children in Birmingham was judged to be inadequate by the UK Office for Standards in Education, Children’s Services and Skills (Ofsted). Ofsted’s assessment highlighted widespread and serious failures that were reported to leave children and young people at risk of harm. The national press has reported number of high-profile child deaths in Birmingham since 2003.

Birmingham, like many other cities, is responding to the Open Data agenda, that is, publishing data and statistical summaries on a variety of topics including school admissions, voluntary and community sector funding, use of car parking spaces and even gritting routes.

In contrast to this, this research uses so-called closed agreements from the Council’s CareFirst System, the primary information system for recording social care provision for all vulnerable children and adults. An agreement here refers to the commissioned delivery of a social service following an assessment of an individual’s needs. An initial analysis of CareFirst in March 2015 showed that the total number of client records exceeded 560,000.

The aim of this research - conducted as part of Birmingham City Council’s Future Council Programme - was to investigate:

- How data held in local authority systems could be analysed and, in contrast to national big- and open-data programmes, provide significant value and insight to in-house local government teams;
- The extent to which data value is impacted when personally identifiable attributes are retained;
- How the use of local authority data could inform future planning and service delivery in Birmingham, as part of the authority’s business planning and budget setting processes.

The remainder of this paper is organised as followed: Background research related to data challenges and their application to social services is presented in Section 2; Section 3 introduces the data sets used in this study and the pre- processing steps necessary to aid analysis. The application of spatial-temporal analytical tools and techniques to the data are documented in Section 4 and, based on this analysis, Section 5 presents the first of three case studies, investigating the impact of a new contractual framework on the delivery of services supporting older adult care. Section 6 documents a second case study, the analysis of existing and future costs of residential respite care for disabled children; the third case study, the internal and external delivery of older adult care in response to the Council’s policy on maximising the independence of adults, is presented in Section 7. Sections 8 and 9 discuss the implications of this research, conclude the paper and detail avenues for further research.

## Background Research

There is an increasing body of work in the public sector related to Big Data and Open Data and, in particular, how these paradigms could assist in transforming public services. The UK Government Open Data White Paper [4] described the United Kingdom as a world leader in the public dissemination of data, citing more than 9,000 datasets that were already available through public portals. It is argued that data are a powerful raw material necessary for holding governments to account, driving decision making and improving the transparency of public services.

In 2011, McKinsey Global Institute published a report on Big Data [5], stating that the capture, curation, search, analysis, visualization and storage of large and complex data sets would generate value across stakeholders in five key domains: health care, public sector administration, retail, global manufacturing and personal data.

A 2012 report by the Policy Exchange [6] argued that applying the technologies of big data alone was insufficient for city transformation and that, as a minimum, data quality and standards needed to be addressed. Nevertheless, their research estimated that performance improvements could result in public sector savings of between £16 billion and £33 billion per annum.

Whilst the benefits of big data and open data are apparent, there is widespread recognition that in exploiting data, organisations may leave themselves vulnerable to breaches in privacy or data exploitation. The issue of realising the benefits of big data, whilst preventing privacy abuses, has been the subject of two reports published by the White House and analysed by PwC [7]. In these reports it was suggested that in order to manage expectations, changes were needed in legislation and a wider recognition of issues was needed within organisations. Thus, the use of data and corresponding issues of privacy need to be integrated into the business strategy of local governments to enable ownership, oversight and benefit, whilst ensuring individuals retain protection to prevent abuse and discrimination.

Matters of privacy and organisational responsibility also feature in work by David Rhind [8], who cites five data protection categories in this context: personal privacy - in which citizen’s information must be kept in confidential; the appropriate role of the state - in disseminating findings appropriately and avoiding misuse; the cause and effect of technology - including risk of data transfer and processing; the lack of quantitative skills - which may impact analysis and, the misrepresentation of scientific findings.

One of the additional challenges facing those wishing to use information relating to individuals held by government, is the uncertainty surrounding the extent to which data held and published can be used for comparative or analytical purposes. A recent study by the Childhood Wellbeing Research Centre, investigated the availability and comparability of statistics related to the safeguarding of children in the UK. This research highlighted divergence in the characteristics of children registered for children’s social care across the country, caused in part by a variation in age and ethnicity groups within the published statistics from different areas of the UK. Likewise, a study by Craglia et al. [9] encountered the issue of data uncertainty for Child Service Plans for Sheffield. Their research found that only half of the data sets supplied by partner organisations met the granularity requirements needed for their analysis.

There is evidence of the use of deidentified data held by government to support service delivery and planning, particularly in relation to vulnerable children [10—12]. Guralnick [13] stated that a well-organised system of early intervention could prevent cognitive impairment in children up to the age of five. In New Zealand, research has been carried out on the use of administrative data [14] in identifying children at risk. This research proposed using data to support predictive risk modelling as a means of tackling issues of child protection and maltreatment. The study highlighted that whilst modelling could identify instances of abuse and neglect, the approach was not without risk of stigmatising and discriminating against certain individuals and families.

Thomas and Percy-Smith [15] take a different approach, citing the effective participation of children and social workers for service planning and provision. They note that the voice of young people who were recipients of these services can be very important and could help shape the overall strategy of services within local areas.

The dual challenges of the big- and open-data agenda, and the need to protect individual privacy, form the backdrop to the research carried out in this study. Firstly, the research aims to highlight the value that can be gained from datasets derived from deidentified data held by a local authority, data which would not normally be subject to further analysis in a more traditional open-data setting. Secondly, in focussing the research on a specific domain (social care provision), it aims to demonstrate that by identifying key data within an organisation, and employing state-of-the-art data analytic techniques, future planning and service delivery within a city can be improved, without the need for additional and costly business analytic services.

We note that this research does not (yet) seek to identify risk factors in specific individuals, rather it aims to support the organisation in understanding where previous demand for services has been met, by type of user and at what cost, in order to support service and budgetary planning in future years. 

## The Data

### Care service agreements

There are three forms of data used in this study, unstructured, semi-structured and structured. All data derives from closed agreements (care services which had been agreed upon, commissioned and delivered by BCC or a third party) which have been extracted from Birmingham City Council’s CareFirst information system. CareFirst has been in operation for over a decade, but includes data records of service deployments dating back much longer than this - initially records for more than 260,000 individuals were extracted, dating back to 1990. The results presented here are for CareFirst closed agreements for the period 2001 to 2015, inclusive.

The data sub-sample included over 31,610 distinct people registered for a total of 119 unique council services, and 360 unique elements (a service is comprised of number of different elements, which may or may not be enacted as part of a delivered service agreement). Each closed agreement consists of a number of attributes, see Table 1.

**Table 1: Records comprising a Closed Agreement and their description; note, only 14 of 18 available records are used in this study. Free-text fields are also present and not listed below.** 

Record	Description
ADE_ID	Agreement ID
PERID	Person ID
DOB	Date of Birth
Agreement Start	Start date of the agreement
Agreement End	End date of the agreement
Service name	Alphanumeric coding of the service
Service Description	Description of the service
Element	Alphanumeric coding of the element
Element Description	Description of the element
Postcode	Postcode at unit level
Gender	Gender status
Ethnicity	Ethnic classification (using census categories)
Disability	Disability status
Weekly Cost	Weekly cost per one agreement element

The Element name is typically stored as a string comprising five or more characters representing a short version of the full element description. A simple coding strategy is employed: An element name that begins with CH is related to children; DIR represents a direct payment; HSSU represents home support; LD is related to learning disabilities; MH is related to mental health; OA refers to a service element for an older adult; PD represents a service for people with physical disabilities and, SM represents a service connected to substance misuse. Table 2 provides example service elements and their description.

**Table 2: Sample service elements and their description.** 

Element Name	Element Description
CHEFODIS	Children, External, Fostering, Disabled
DIRCWD	Direct Payments, Children with Disabilities
HSSU65PL	Home Support, 65 Plus, External Community Based
LDEHSQDS	Learning Disability, External, Quick Discharge Service
MHEBLACT	Mental Health, External, Block Activity
OAICINT	Older Adults, Interim Care, Internal
PDEHSUPP	Physical Disabilities, External, Supported Living

The total value of the expenditure of service agreements extracted from the system was estimated at slightly above £670 million for the sample period in question. 

### Data cleansing and data-processing workflow

Spatial-temporal matrices were developed to explore data quality and conduct anomaly detection. Our analysis began with postcode districts (rows) and the years 2001 to 2015 (columns) and built a frequency table of the number of registered agreements for each district in each year. Data outside these geographical and temporal boundaries were removed and colour gradation (green to red) was used to highlight those areas with higher concentrations of registered agreements. Given the quality and concentration of records, data were selected for this research from the period 2010 to 2015.

The data were further categorised into four age groups according to Council norms: Children aged 0-11; Young people and adults, aged 11-25; Adults aged 25-65 and older people, aged 65-90. Records were retrieved for these age ranges, see Table 3; we note that there will be some duplications of individuals with such categorisation, as a person may be registered for more than one service within a year.

**Table 3: Number of service agreement records with respect to the four age groups.** 

Age Category	Number of Records (approx.)
0-11	7,300
11-25	26,000
25-65	47,000
65-90	133,000

**Figure 2: Workflow employed in this research.**
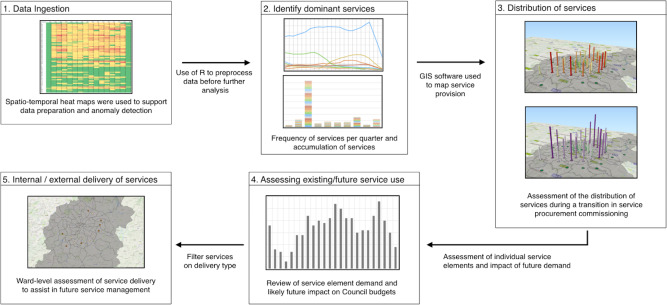


We illustrate our data-processing workflow in Figure 2 . Data ingestion, cleansing and anomaly detection are depicted in stage 1. Pre-processing scripts and the statistical tool R are used, removing erroneous characters, conducting range checks and identifying missing values. Of the 258,673 closed agreements studied, 18,872 (7.3%) are removed because of ‘bad data’: The majority of the cases involved (i) missing values, (ii) unreadable or invalid data records, (iii) unknown or invalid age entries and (iv) unknown gender.

In stage 2 we analyse all 119 services and 360 service elements to understand which services dominate in terms of both cost and frequency. The analysis is typically presented per quarter and captures the cost and frequency at each quarter as well as the accumulated cost and frequency per service element.

In stage 3 we employ open-source geographical information systems to perform the spatial-temporal mapping. Postcodes in the closed agreements (alphanumeric identifiers of six to eight characters, which designate an area with a number of distinct addresses) comprise a postcode area and postcode district (the outward code) and a postcode sector and a postcode unit (the inward code). The exploration was possible at the sector level, at which point the data were spatially joined with a geographic shapefile (in the ESRI vector data storage format) representing the location, shape and attributes of the corresponding geographic unit. Coordinates are plotted using the Ordnance Survey National Grid reference system (BNG) with the European Petroleum Survey Group (EPSG) Code EPSG:27700. Plugins for Google Maps and OpenStreetMap (OSM) are employed from the QGIS OpenLayers Plugin.

We elaborate on stages 4 and 5 below. Stage 4 corresponds to the second of our three case studies, where the frequency of service elements is analysed and, together with population data, predictions are made as to the likely increase in demand (and cost). In stage 5, ward-level assessment is conducted to assist the Council in business planning and budgetary objectives in relation to the policy of Maximising the Independence of Adults (MIA).

## Spatial-Temporal Analysis

Temporal analysis of the data is performed: (i) as a single sample period 2010-2015 (6 years in total); (ii) in two three-year sample blocks, 2010-2012 and 2013-2015 and, (iii) quarterly, resulting in 24 consecutive time periods for the sample in question. Analysis of services and service elements is based on seven of the CareFirst data records and the analysis of users is based on nine of the CareFirst data records, see Table 4. The analysis focusses on the top ten service elements, the top three, accounted for about 12% of the total expenditure, of which are explored further in the subsequent case studies.

### Identifying the top ten service elements over time

**Table 4: The CareFirst data records utilised in subsequent case studies in this paper.** 

Variable Name	Variable Type
Postcode	Spatial
Coordinates	Spatial
Agreement Start	Temporal
Agreement End	Temporal
Element Name	Temporal
Element Description	Temporal
Weekly Cost	Temporal
Age	Spatial and Temporal
Ethnicity	Spatial and Temporal

**Figure 3: Top ten service elements in terms of cost, for all age categories for the period 2010-2015.**
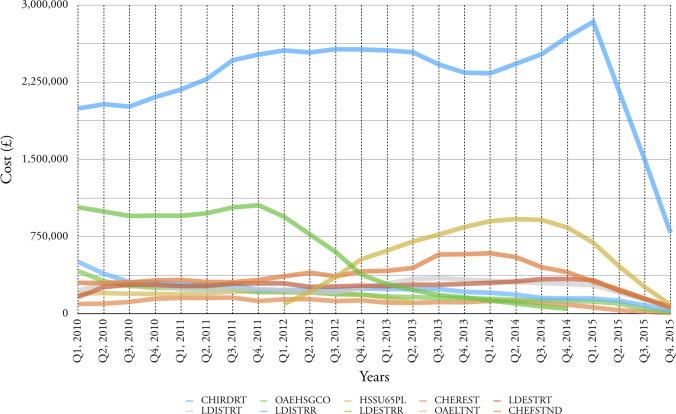
(a) Cost of service per quarter

**Figure 3: Top ten service elements in terms of cost, for all age categories for the period 2010-2015.**
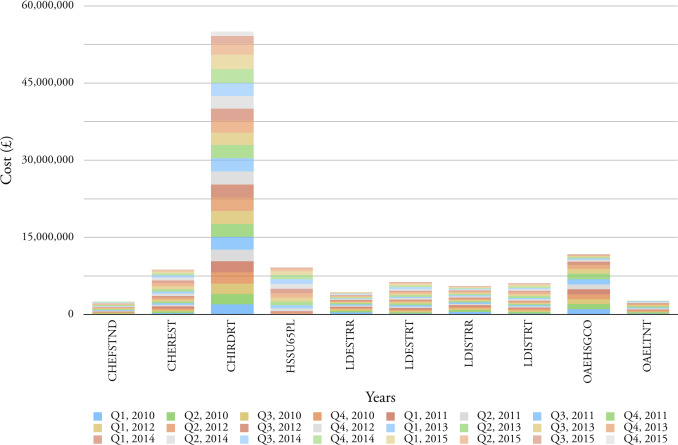
(b) Accumulative cost for each service element

**Table 5:Top ten service elements in terms of cost for the period 2010-2015.** 

Service Element	Element Description	Cost
CHIRDRT	Children Internal Residential Disabled Respite	£54,964,000
OAEHSGCO	Older Adults External General Contracted	£11,864,117
HSSU65PL	Home Support 65 Plus External Community Based	£9,206,837
CHEREST	Children External Residential Home	£8,823,100
LDESTRT	Learning Disability External Short Term Residential	£6,407,018
LDISTRT	Learning Disability Internal Short Term Residential	£6,172,000
LDISTRR	Learning Disability External Short Term Residential	£5,561,040
LDESTRR	Learning Disability Internal Short Term Residential	£4,453,364
OAELTNT	Older Adults External Long Term Nursing	£2,697,419
CHEFSTND	Children External Fostering Standard Fee	£2,609,469

Total		£112,758,364

Figure 3a shows the top ten service elements in terms of quarterly cost and the fluctuation in this cost over six years. The service element CHIRDRT (Children Internal Residential Disabled Respite) dominates and shows a steady increase in cost between Q1_2010 and Q1_2015; Figure 3b reports that this service element accounts for £54,964,000 over the six years in question. Table 5 documents the cost of the top ten service elements for the period 2010-2015; of these, more than half are delivered by external providers.

**Figure 4: Top ten service elements in terms of commissioning frequency, for all age categories for the period 2010-2015.**
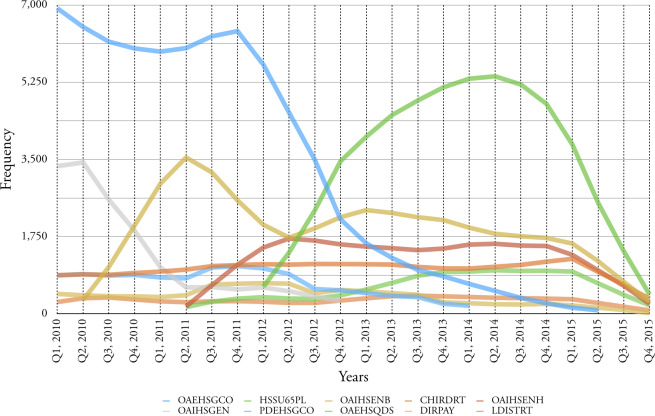
(a) Frequency of service per quarter

**Figure 4: Top ten service elements in terms of commissioning frequency, for all age categories for the period 2010-2015.**
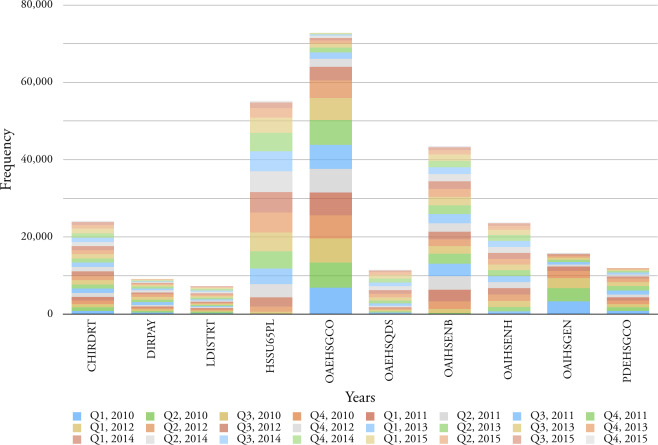
(b) Accumulative frequency for each service

**Table 6: Top ten service elements in terms of frequency for the period 2010-2015.** 

Service Element	Element Description	Frequency
OAEHSGCO	Older Adults External General Contracted	72,860
HSSU65PL	Home Support 65 Plus External Community Based	55,199
OAIHSENB	Older Adults Internal Home Support Enablement	43,524
CHIRDRT	Children Internal Residential Disabled Respite	24,160
OAIHSENH	Older Adults Internal Home Support Enhanced Assessment	23,743
OAIHSGEN	Older Adults Internal Home Support General	15,875
PDEHSGCO	Physical Disability External General Contracted	11,984
OAEHSQDS	Older Adults External Quick Discharge Service	11,500
DIRPAY	Direct Payments	9,217
LDISTRT	Learning Disability Internal Short Term Residential	7,348

Total		275,410

In addition to cost, Birmingham City Council is also interested in the commissioning frequency of service elements as each commission requires associated administrative overhead. In Figure 4a we document the top ten service elements in terms of quarterly frequency and the fluctuation in this commissioning frequency over six years. Table 6 clearly shows that the amount of care services are provided to older adults more than the younger group over time.

This analysis raises number of questions, but we restrict our discussion to four services:

1. OAEHSGCO and HSSU65PL, represent older-adult care and rank top in terms of frequency and in the top three services in terms of cost over the period in question. There is an interesting connection between these service elements due to the introduction of a new contractual framework for the procurement of home support services. In our first case study, we explore and highlight this pre- and post-contractual change and its impact on the city;
2. CHIRDRT, Children’s Internal Residential Disabled Respite, is the most costly service to the Council, accounting for £55 million over the period in question. Despite the cost, and associated frequency, this service is used by a relatively small number of unique registered users, as our second case study will explore;
3. OAIHSENB, is an internal service delivered directly by the Council to support the care of older adults. Whilst this service does not feature highly in terms of cost, it is however an internally managed service commissioned over 43,000 times during the period in question. Case study 3 investigates the geographic areas within the city in which this service is most used to better understand ongoing and future management.

Before we begin the three case studies outlined above, we highlight two (of many) anomalies that this exploration of data exposes. Two service elements are chosen to illustrate our findings: CHERSET - denoting that a child is/was placed at an external residential setting as a result of their assessment needs and, LDELVRT - denoting a variation in contract, possibly following a review, of a long-term residential placement to support the learning disabilities of a child. For each we provide quarterly cost for the period 2010 to 2015, see Figure 5a and Figure 5b The predictability of the provision of these two service elements is highly variable: The cost per quarter ranges from £0 to approximately £180,000; There are extended periods when these service element codes are not used at all; There are outliers which make the financial management of these service elements difficult; and there are features in the data which echo responses to priorities, care service management and financial pressures.

**Figure 5: Example of anomalies that the exploration of data expose.**
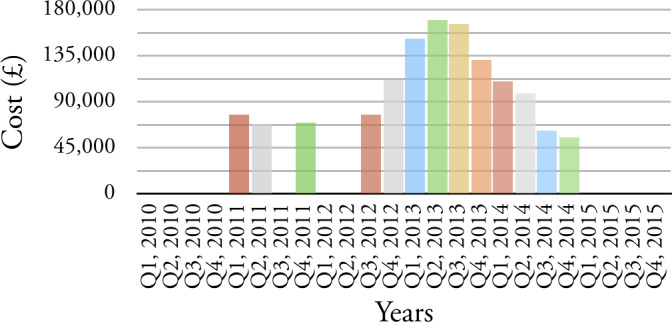
(a) CHERSET

**Figure 5: Example of anomalies that the exploration of data expose.**
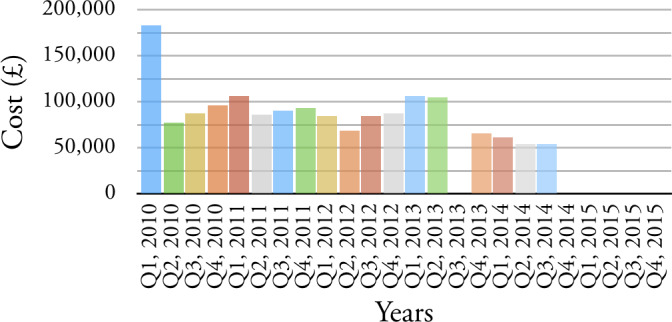
(b) LDELVRT

**Figure 6: Annual cost of top twenty elements of six-year period.**
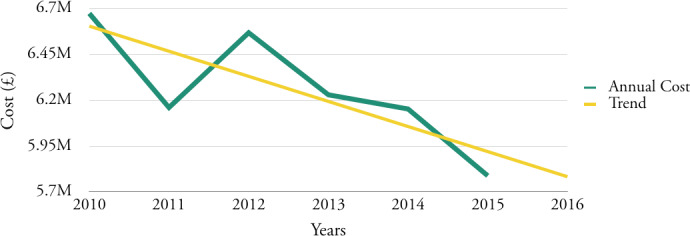


Financial analysis of the top twenty service elements also highlights that the annual cost of these has, in general terms, fallen during the period 2010 to 2015, see Figure 6 . Given the Council’s budget reduction plan, this trend is likely to continue.

## Case Study 1 - The impact of a new contractual framework

Our first case study sought to understand the transition of the delivery of services following the implementation of a new contractual framework. Particularly, we were interested in understanding whether this service transition impacted some of Birmingham residents more than others.

The change in contractual management of the agreement which we highlight came about as a result of The Adults and Communities Transformation Programme Future Operating Model identifying the need for a different approach to blocking contract purchasing of adult social care provision. Following a tender in 2011, the People Directorate commissioned a micro-procurement system (Sproc.net) to procure individual home support and bed-based care packages for Birmingham citizens with eligible needs.

In 2012, Sproc.net became the procurement system of choice for home support commissioning and, in October 2013, the system was extended for older adults’ nursing and residential care.

We focus on the service elements OAEHSGCO and HSSU65PL identified earlier, both of which relate to older-adult care and which fall under the 2011/12 transformation programme. We show the geographical dispersal of the service elements and service users. Data are aggregated over a six-year period (2010-2015) and two colour ramps are used for each graphical cylinder - purple to green for the service element OAEHSGCO (Figure 7a), and red to green for the service element HSSU65PL (Figure 7b). The height of each cylindrical bar is determined by the number of individuals registered within the specific postcode; a higher bar indicates that more agreements have been made. Note that multiple agreements for the same service, for a unique individual, will only register once.

**Figure 7: Geographical dispersal of the OAEHSGCO and HSSU65PL service elements across.**
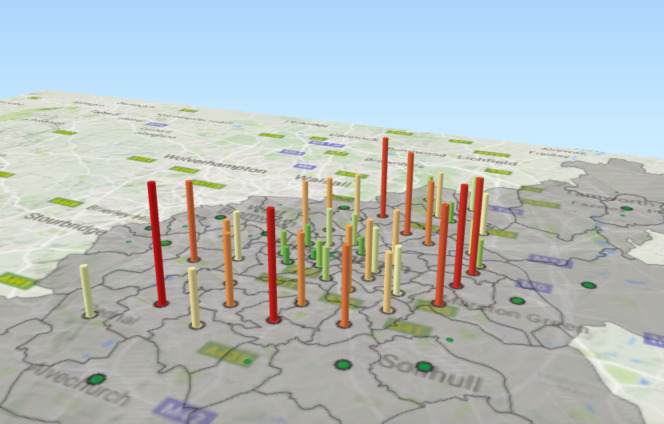
(a) HSSU65PL

**Figure 7: Geographical dispersal of the OAEHSGCO and HSSU65PL service elements across.**
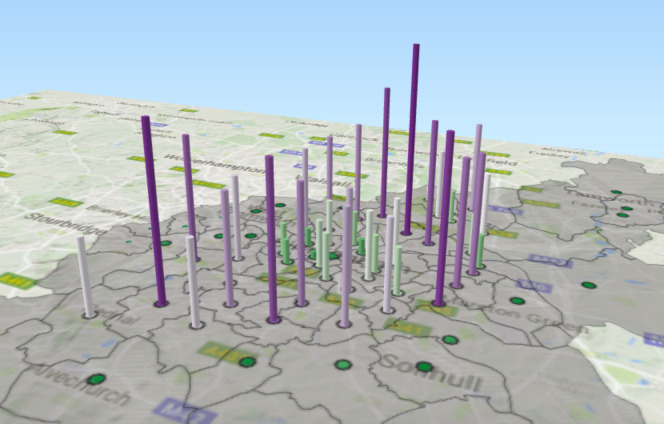
(b) OAEHSGCO

**Table 7: OAEHSGCO and HSSU65PL agreements for difference postcodes in Birmingham.** 

Postcode	OAEHSGCO	HSSU65PL	Total	Difference
B23	1,254	564	1,818	-690
B24	816	426	1,242	-390
B75	645	261	906	-384
B26	952	569	1,521	-383
B42	690	332	1,022	-358

Table 7 highlights the five postcodes where the difference between these two delivered services was the greatest (note that one might expect the frequency of OAEHSGCO and HSSU65PL to remain consistent as the Council transitioned from one service delivery framework to the other). The areas with the largest drop in unique service delivery (before and after the Sproc.net service delivery transition) are clustered in the northern part of the city (postcodes B23, B24, B75, B42); conversely, the areas with the greatest increase in the number of agreements is in central Birmingham (postcodes B9, B18, B7, B3, B1); note these are not shown in Table 7 .

According to the 2011 census, the distribution of ethnic groups in Birmingham is mixed. Postcodes B23 and B24, for example, have a population which is 77.9% white; B9 on the other hand has a white population of just 27.1%. We were interested therefore in whether certain ethnic communities were impacted more by the transition of older adult care than others, as the demographics of the populations of the regions most affected might suggest.

Figure 8 displays the distribution of ethnicity for the recipients of OAEHSGCO and HSSU65PL as pie-charts. There are six out of a possible twenty ethnicity groups included within the agreement datasets. A large majority of the registered users are White, followed by Asian, Black, Others, Mixed Parentage and Not Given (information not obtained). These data are somewhat reassuring: Whilst it is clear that the service framework transition has impacted the establishment of new agreements, particularly in the north of the city, this impact is not however limited to one ethnicity group more than any other. Clearly, there is work to do in understanding why the service framework transition has had such an effect, and supporting research will be needed to establish procedures to mitigate for similar issues in the future.

**Figure 8: Ethnicity profile of the recipients of OAEHSGCO and HSSU65PL.**
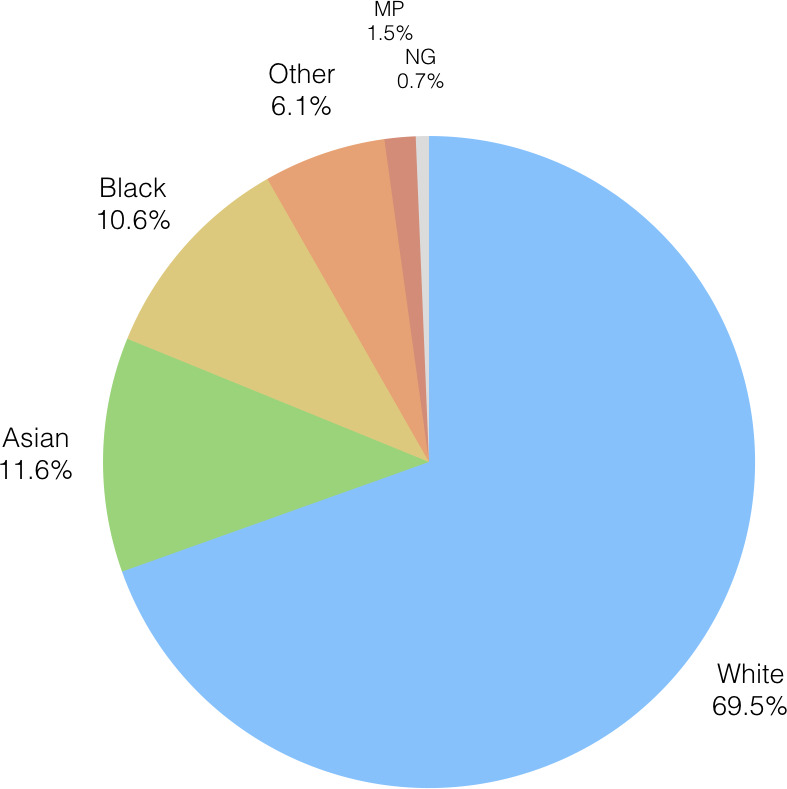
(a) Ethnicity profile HSSU65PL

**Figure 8: Ethnicity profile of the recipients of OAEHSGCO and HSSU65PL.**
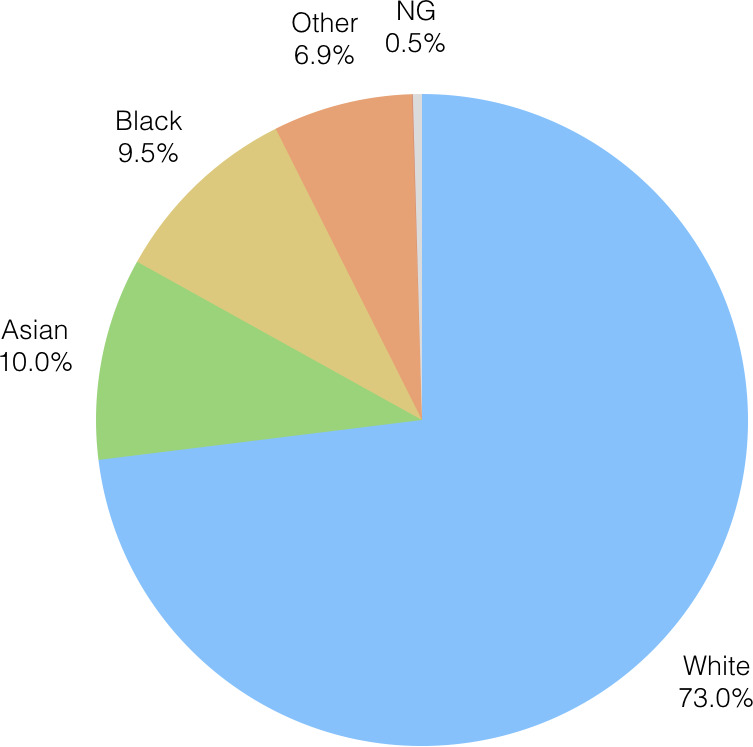
(b) Ethnicity profile OAEHSGCO

## Case Study 2 - Residential respite care for disabled children

**Figure 9: Ethnicity profile of the recipients of OAEHSGCO and HSSU65PL.**
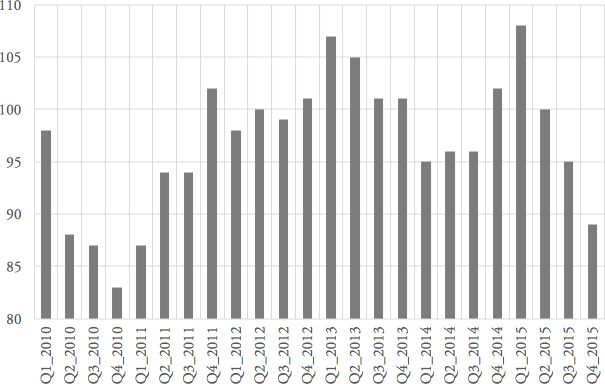


Residential respite care for disabled children (CHIRDRT) accounts for approximately £55 million of Council’s spend on care services over the six-year study period. This service element dominates the spend profile (Figure 4a) and shows a steady increase in cost between Q1_2010 and Q1_2014. Despite this steady rise in cost, the number of unique registered users for CHIRDRT varies significantly over the same period, as shown in Figure 9.

The number of unique registered users in each quarter varies by as much as 20%. Unlike the first case study, in which service elements had a significant cost and frequency, this case study highlights that although the cost and frequency of service agreements are also high, the number of service users is comparatively small, see Table 8.

Interestingly, we also find that a high proportion of the users in Q1_2010 are not found in Q1_2015. There are several explanations for this, including (i) that the user drops out of the 5-18 age bracket caught by this service element code (we see around 22% of users transition to the adult age category in our dataset) and (ii) that users no longer live in the area.

**Table 8: OAEHSGCO and HSSU65PL agreements for difference postcodes in Birmingham.** 

Condition	Q1_2010	Q1_2015
Number of unique registered users	98	108
Number of unique users between Q1_2010 and Q1_2015	76	86

It is clearly advantageous to attempt to predict this population in Birmingham when modelling service expenditure in future years. Birmingham has the youngest population of any European city and, according to population estimates and census data, more than 28% of the Birmingham population are aged under 19. The Birmingham City Budget Plan 2016 provides more detailed population statistics: Between 2001 and 2011, the 0-4 age group grew by 17% and now accounts for 7.8% of Birmingham’s population. This growth is set to slow to 1.1% between now and 2021; the largest growth will be the 10-14 age group, which will see its population increase by 7.7%.

This presents several challenges to the Council, as the number of people in Birmingham eligible for care services such as CHIRDRT grows. The current population projections point to, we believe, an increase in demand for residential respite care for disabled children and we expect the cost of this service element to increase by around 15% between now and 2021, if the cost of this service remains static.

## Case Study 3 - Care services for older adults

The third case study, in contrast to the previous two, focuses on the delivery of older adult care. The Council provides housing support and enablement for older adults both through a commissioned service from external providers (a) OAEHSCGO (Older Adults External General Contracted) and (b) HSSU65PL (Home Support 65 Plus External Community Based) and as a provider of the service itself (c) OAIHSENB (Older Adults Internal Home Support Enablement).

As a result of the Council’s budget setting proposals for 2015/16, which considered externalisation of existing internally provided services, the Council wanted to understand if the provisioning patterns were similar for the internal and external services or, if there was significant deviation between these, where and to what extent this manifested itself in the city.

**Figure 10: Postcode regions which saw the highest concentration of the three service elements in question between 2010 and 2015: Darker spots, 2 standard deviations above the mean; Lighter spots, 1 standard deviation above the mean.**
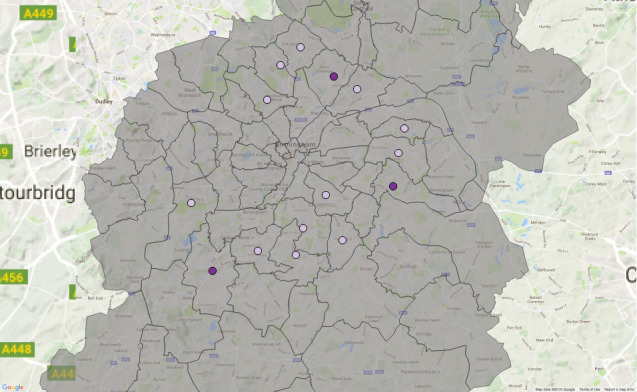
(a) OAEHSGCO

**Figure 10: Postcode regions which saw the highest concentration of the three service elements in question between 2010 and 2015: Darker spots, 2 standard deviations above the mean; Lighter spots, 1 standard deviation above the mean.**
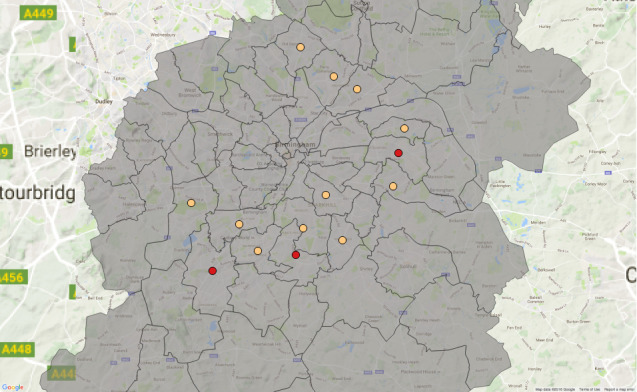
(b) HSSU65PL

**Figure 10: Postcode regions which saw the highest concentration of the three service elements in question between 2010 and 2015: Darker spots, 2 standard deviations above the mean; Lighter spots, 1 standard deviation above the mean.**
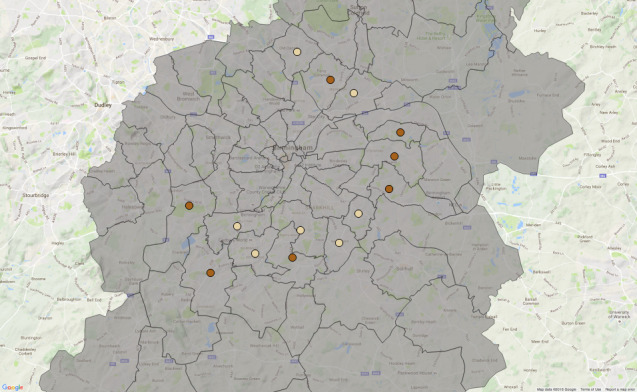
(c) OAIHSENB

Figure 10 shows the concentration of the three service elements in question between 2010 and 2015. The ‘outer ring’ pattern is clear for all three service elements and indeed there appears little difference in the externally provided service HSSU65PL and the internally provided service OAIHSENB.

**Figure 11: Density plots comparing the three service elements over the period 2010 to 2015.**
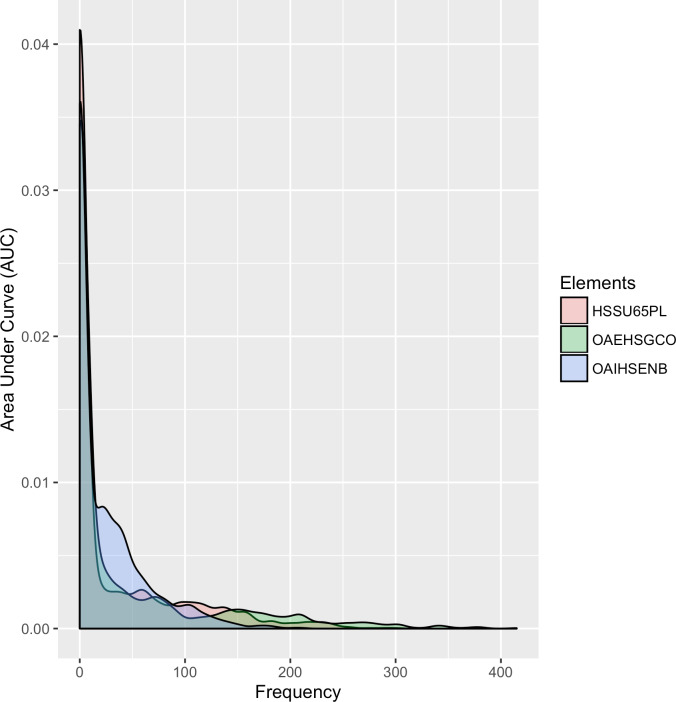


In order to verify these findings, we calculate the distribution frequencies for all three service elements for all 75 postcode regions. Our analysis considers all calendar quarters of a six-year period (2010-2015). Figure 11 shows the distribution of frequencies within the three service elements over a continuous interval in density format.

Figure 11 highlights that the two external elements (OAEHSGCO and HSSU65PL) show similar trends, with a sharp descent and a narrow tail, indicating that few postcode regions have large number of registered agreements. The peak of the curve helps us to identify where services are concentrated and at what frequency. The area under the curve for the internal element shows a higher density of service provision in some postcode areas, typically where between 20 and 70 service agreements are delivered. This confirms the findings in Figure 10 , but allows us to tune our conclusions accordingly.

With such analysis, it is possible to be very accurate in calculating the similarities (and differences) in service element delivery. This pattern identification will be used to support the development of commissioning strategies for externalising internal services.

## Discussion

The starting point for this research was to consider how social care data already held by Birmingham City Council could be extracted, analysed and used to support decision making considering the financial challenges facing the local authority. This study focuses on the authority’s social care system, CareFirst, although other similar data exist in the council and many other opportunities exist in this regard. To begin the process of accessing the data, the researchers followed and gained approval for the research through the council’s internal governance processes to ensure compliance with relevant data protection and ethical obligations. All data were deidentified before receipt (all identifiable attributes were removed), so that it was not possible for the researchers to identify individuals or groups of individuals.

CareFirst is the council’s primary case management system used for social care referrals, assessments and the recording of service agreements. Our workflow began with data extraction, ingestion, cleansing and spatial-temporal analysis to derive a data model suitable for further analysis and manipulation.

Three case studies were selected to demonstrate how research and insight can be obtained from data held within local authority systems, through a targeted evaluation of the data alongside historical records of service management frameworks and key Council priorities and objectives. Several attributes of the data, including anonymised person IDs, commissioning dates, approximate location and service costs are critical to understanding the provisioning of social care services and the trends and demands that these services are subject to over time.

The primary purpose of data collection within the CareFirst system is the delivery of services and the management of caseloads, as opposed to supporting analysis and research, and making use of the data beyond its original purpose is challenging. However, as this research shows, with the support of suitable anonymisation and data analytic techniques, data are assets that local authorities may increasingly look towards to support budget reduction challenges whilst supporting and maintaining levels of service to a diverse population.

The use of postcode sector data and individual attributes raises questions of data protection and privacy. As described earlier, the data are extracted from the Council’s social care system, and to comply with the provisions of the Data Protection Act 1998 (the Act), the data are depersonalised at source (i.e. before being made available for research) to prevent the identification of any individuals or groups. This allows the researchers (and the Council) to demonstrate compliance with Section 33Section 33 of the Data Protection Act (1998) has been repealed and replaced by Section 19 of the Data Protection Act (2018) of the Act provided that: *“(a) the data are not processed to support measures or decisions with respect to specific type of individuals, and (b) the data are not processed in such a way that substantial damage or substantial distress is, or is likely to be, caused to any data subject.”* Furthermore, the Act states that: *“the further processing of personal data for research purposes in compliance with conditions, (a) and (b) above, is not to be regarded as incompatible with the purposes for which it was obtained.”* It is within the parameters of these conditions, and under the jurisdiction of the Councils ethics and governance procedures, that this research is conducted.

Whilst the Act clearly defines parameters for our research, our study falls into a common class of problem - the desire to understand aggregate information about data, without exposing data about individuals themselves. This problem is well understood in the context of population census studies (the 2016 Australian census was criticised for this very reason [16]) and as a result, an emerging collection of methods, including differential privacy [17], have been developed to ensure anonymisation in large sparse datasets.

Whilst there are risks associated with the use of even deidentified data, it should be recognised that, if appropriately utilised and by following relevant legal, ethical and organisational requirements, the data can provide evidence of continuity of service and public good, and improve the operations of public services in the UK and beyond. The retention and use of data in Case Study 1 are used to demonstrate that following the implementation of a new contractual framework, the ethnic mix of recipients of this service remained largely consistent, even if the delivery of services in some areas did drop.

As each of the three case studies highlights, our analysis allows past provisioning of services to be better understood, trends in the delivery of services to be identified and, future demand to be forecasted.

Each case study has a different focus, demonstrating varied capability. Case study 1 investigates the impact on older adult care of transitioning from one contractual framework to another, identifying those postcodes which may have been impacted by this transition. Case study 2 considers services provided to disabled children, a small pool of recipients aged between 5 and 18. Respite services are costly, and we show how it is possible to model the likely increase in these costs in future years. Case study 3 explores how CareFirst data can be used to understand the relationship between the provision of services provided by an external provider and those provided directly by the Council. This work will also support the Council in its aim to save around £9 million on Home Care Enablement between now and 2020.

### Conclusions and Future Work

This research employs state-of-the-art data analytic techniques to analyse six years of Birmingham City Council social care data, to identify: (i) Service cost profiles over time; (ii) Geographic dimensions to service demand and delivery; (iii) Patterns in the provision of services, which may assist with future service planning and provision, and (iv) The extent to which data value and data protection interact.

Data used in this research derives from Birmingham City Council’s CareFirst information system. The data sub-sample used included over 31,610 distinct people, registered for a total of 119 unique council services and 360 unique service elements; representing the delivery of 258,673 social care services. Heat maps were developed to explore data quality and conduct anomaly detection; 18,872 closed agreements (7.3% of the total) were removed from our study because of bad data. Temporal analysis of the data allowed us to identify the top ten service elements over time, according to (i) cost and (ii) frequency of delivery; the total value of these top-ten services exceeded £112 million for the period 2010 to 2015.

Spatial-temporal data analysis highlighted several service anomalies and focussed on three case studies: The impact of a new contractual framework on the older-adult home support; Residential respite care for disabled children, and care services for older adults. All three case studies demonstrated how data held in local authority systems could be exploited and, in contrast to national big- and open-data programmes, provide significant value and insight to in-house government teams. This research aims to inform future planning and service delivery in Birmingham, as part of the authority’s business planning and budget setting processes.

This research is continuing by looking at the earlier stages in the service provision pipeline - notably the pre-assessment and assessment stages for older adults seeking access to Council services. Future research will focus on the journey of service users from referral, contact assessment, assessment, development of a support plan and the subsequent delivery of the service agreement. The research will aim to understand the levels of demand for access, the process that the service users undertake and, as with this research, anomalies and variations in the quality of data.

Birmingham City Council is expected to make savings of £815 million over the nine-year period 2011/12 to 2019/20. Delivering savings of this scale, whilst protecting and safeguarding the most vulnerable citizens within a growing urban population, is one of the biggest challenges facing the UK’s second largest city. Data-led research such as this offers significant opportunity to facilitate and understand such change.

## Acknowledgement

The lead author gratefully acknowledges support by the UK Engineering and Physical Sciences Research Council (EPSRC) for the Centre for Doctoral Training in Urban Science and Progress under Grant number [EP/L016400/1]. The authors also thank Birmingham City Council for providing support and data access through their internship programme.
